# Kynurenine catabolic enzyme KMO regulates HCC growth

**DOI:** 10.1002/ctm2.697

**Published:** 2022-02-20

**Authors:** Zhaopeng Shi, Guifang Gan, Xianfu Gao, Fuxiang Chen, Jun Mi

**Affiliations:** ^1^ Hongqiao International Institute of Medicine, Tongren Hospital Shanghai Jiao Tong University School of Medicine Shanghai China; ^2^ Key Laboratory of Cell Differentiation and Apoptosis of the Chinese Ministry of Education, Basic Medical Institute Shanghai Jiao Tong University School of Medicine Shanghai China; ^3^ Department of Laboratory Medicine Ninth People's Hospital Shanghai Jiao Tong University School of Medicine Shanghai China; ^4^ Shanghai Profleader Biotech Co., Ltd Shanghai China

Dear editor:

Our recent research has found that the kynurenine derivative 3‐HAA was lower in tumour cells due to the downregulation of its synthetic enzyme kynurenine 3‐monooxygenase (KMO), and overexpression of KMO suppressed hepatocellular carcinoma (HCC) tumour formation and tumour growth by increasing endogenous 3‐HAA. It is well known that kynurenine promotes tumour growth by directly binding to the aryl hydrocarbon receptor.^1^, ^2^, ^3^ The 3‐hydroxyanthranilic acid (3‐HAA), a derivative of kynurenine, was reported to induce apoptosis by upregulating phosphatases.^4^ However, the metabolism and function of kynurenine derivatives largely remain unclear. Here, we report our novel findings related to kynurenine metabolism.

**3‐HAA is decreased in tumour cells**. Tryptophan catabolites were first analysed in clinical HCCs. The concentration of kynurenine catabolite 3‐HAA decreased in both HCC and oesophageal carcinomas compared to the matched paratumour tissues (Figure 1A; Figure S1A). Conversely, the concentration of tryptophan and kynurenine was higher in these HCCs and oesophageal carcinomas than in the matched paratumour tissues, respectively. Consistent with this observation, the concentration of 3‐HAA was also lower in seven HCC cell lines tested than in normal hepatic cells, whereas the content of tryptophan and kynurenine increased in these tested HCC cell lines (Figure 1B). The immunohistochemistry analysis further confirmed lower 3‐HAA content in clinical HCC tissues than in adjacent non‐cancerous tissues (Figure 1C).

**FIGURE 1:**
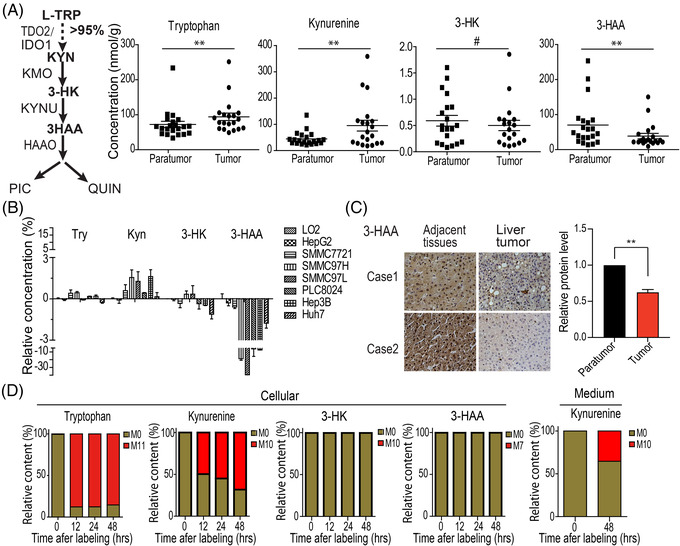
3‐HAA is decreased in tumour cells. (A) Quantitative analysis of tryptophan metabolites by liquid chromatography‐tandem mass spectrometry (LC‐MS/MS) and gas chromatography‐mass spectrometry (GC‐MS) in HCC and the corresponding paratumour tissues. **: *p* < .01. (B) Quantitative analysis of tryptophan metabolites by LC‐MS/MS and GC‐MS in normal hepatic cells and HCC cells. (C) The immunohistochemistry staining of 3‐HAA on HCC samples. (D) Metabolic flux analysis of tryptophan metabolites in SMMC‐7721 and HepG2 cells. l‐Tryptophan was completely ^13^C‐labelled. The content of tryptophan catabolites in cells and medium was assessed using LC‐MS/MS. The M0 stands for no carbon in tryptophan was ^13^C‐labelled, M11 stands for all 11 carbons in tryptophan were ^13^C‐labelled

Metabolic flux analysis revealed tryptophan metabolised to kynurenine but not 3‐hydroxykynurenine (3‐HK) or 3‐HAA in HCC cells, and the newly generated kynurenine was secreted into the culture medium (Figure 1D), suggesting 3‐HAA is decreased in tumours, at least in HCCs and oesophageal carcinomas.

**Upregulation of KMO increases 3‐HAA**. To determine whether the metabolic enzymes regulate 3‐HAA concentration, we assessed the expression of 3‐HAA‐related enzymes in HCC cells. The immunoblotting and immunohistochemistry analysis showed that KMO and kynureninase (KYNU) were downregulated in HCC cells and tissues. In contrast, the indoleamine 2,3‐dioxygenase 1 (IDO1) and tryptophan 2,3‐dioxygenase (TDO2) was upregulated (Figure 2A,B). This finding was consistent with the HCC expression profile in the TCGA database (Figure 2C). Moreover, both KMO and KYNU expression (www.gtexportal.org) are commonly downregulated in tumours originated from tissues abundantly expressing KMO and KYNU. These tumours include lung, kidney, and liver carcinomas, which are the top 10 tumours worldwide in terms of death (Figure 2D).

**FIGURE 2:**
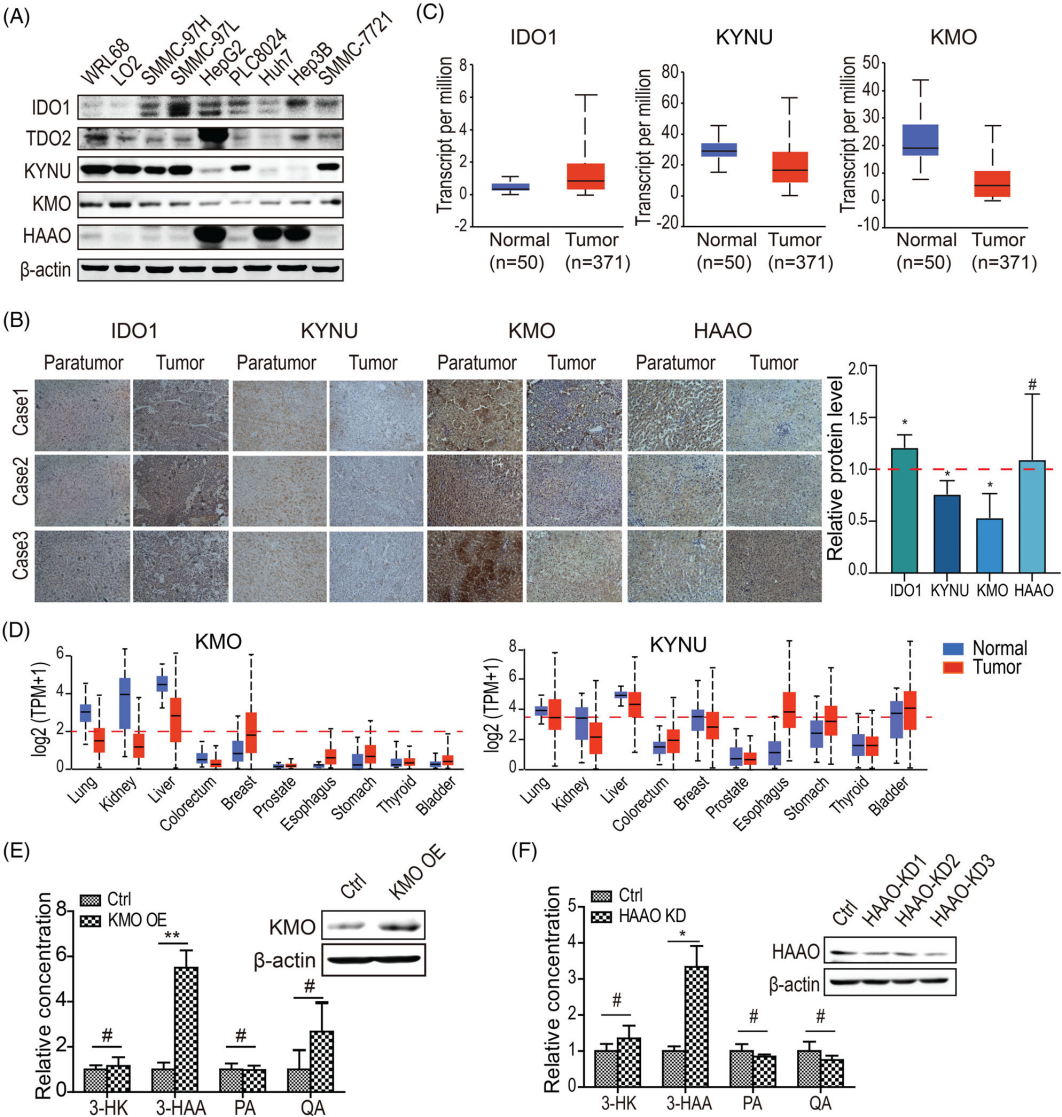
Upregulation of KMO increases 3‐HAA. (A) Expression analysis of metabolic enzymes involved in 3‐HAA generation. HepG2 cells were used. (B) The immunohistochemistry staining of KYNU, KMO, IDO and HAAO on HCC samples. (C) The expression profile of KYNU, KMO, and IDO1 in the HCC samples by RNA‐seq. (D) The expression profile of KMO and KYNU in the top 10 tumours in terms of death and the corresponding normal tissues. KMO: log_2_(TPM) > 2; and KYNU: log_2_(TPM) > 3.5. (E) The effect of KMO overexpression on the concentration of endogenous 3‐HAA and tryptophan catabolites in SMMC7721 cells. **: *p* < .01. (F). The quantitative analysis of 3‐HK, 3‐HAA, PA and QA in SMMC7721 cells depleted of HAAO. *: *p* < .05

In addition, overexpression of KMO significantly increased the concentration of 3‐HAA in HCC SMMC7721 cells, but not the 3‐HK, picolinate (PA), or quinolinate (QA; Figure 2E). The hydroxyanthranilate‐3,4‐dioxygenase (HAAO) knockdown had similar effects on the levels of these metabolites (Figure 2F).

**KMO overexpression inhibits tumour formation by inducing apoptosis**. Functionally, either overexpression of KMO or knockdown of HAAO inhibited cell growth of HCC cells in vitro by increasing apoptosis (Figure 3A,B). Only the apoptosis inhibitor zVAD restored growth of HCC cells following 3‐HAA treatment or overexpressing KMO (Figure 3C,D). Moreover, KMO overexpression suppressed tumour formation and tumour growth in the HCC xenograft nude mice model (Figure 3E; Figure S2A). Remarkably, the Kaplan–Meier survival analysis showed that HCC patients with high KMO expression had a prolonged disease‐free survival than patients with low KMO expression (Figure 3F). The 3‐HAA treatment significantly inhibited HCC cell growth and colony formation (Figure 3G; Figure S2B). Moreover, 3‐HAA but not kynurenine slowed tumour growth in a CDX model and in a patient‐derived xenograft (PDX) model (Figures S2C and 3H), suggesting KMO overexpression inhibits tumour formation and tumour growth via its catabolite 3‐HAA.

**FIGURE 3:**
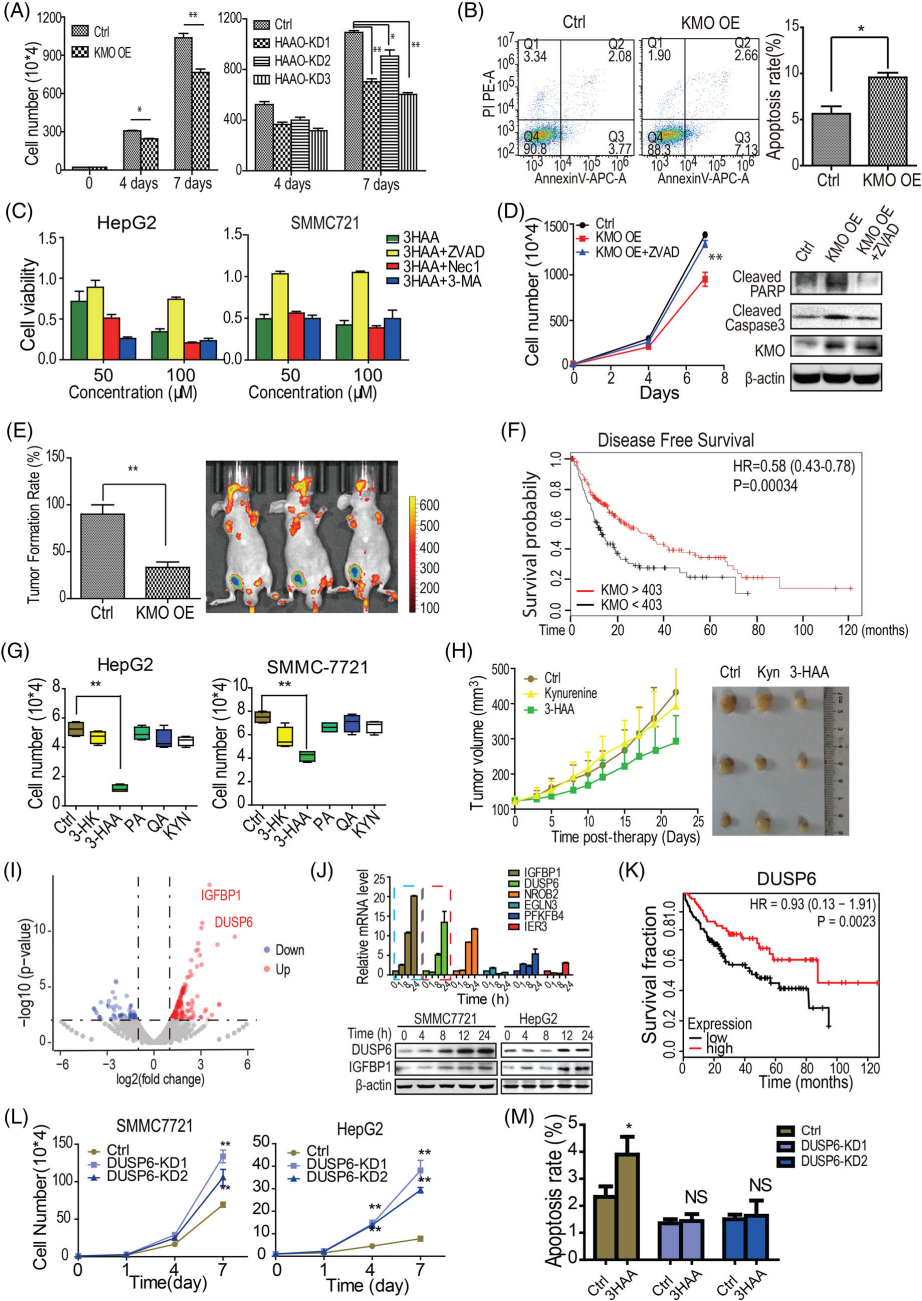
KMO overexpression inhibits tumour formation by inducing apoptosis. (A) The effect of HAAO and KMO on the cell growth of SMMC7721 cells. The KMO and HAAO were overexpressed or knocked down in SMMC7721 cells, separately. *: *p* < .05, **: *p* < .01. (B) The effect of KMO overexpression on apoptosis of SMMC7721 cells. *: *p* < .05. (C) The effects of three types of inhibitors on 3‐HAA‐induced HCC cell death. ZVAD: apoptosis inhibitor (20 μM); Nec1: necrosis inhibitor (100 μM); 3‐MA: autophagy inhibitor (5 mM). The dose of 3‐HAA was 100 μM. Cells were treated for four days. *: *p* < .05, **: *p* < .01. (D) The influence of KMO overexpression on cell growth and apoptotic signal of HCC cells. The KMO was over‐expressed in SMMC‐7721. **: *p* < .01. The dose of 3‐HAA and ZVAD was 100 μM and 20 μM, respectively. (E) The influence of KMO overexpression on tumour formation of HCC cells and the representative living image of HCC xenografts expressing KMO. The KMO was overexpressed in SMMC 7721 (*n* = 10). The cell number for each inoculation was 1.5 * 10^6^. **: *p* < .01. (F). The K_M plot analysis of KMO expression with the disease‐free survival of HCC patients. The patients were divided by the median of KMO expression value. (G) Analysis of the growth of HCC cells treated for 4 days with one of five tryptophan metabolites (100 μM). **: *p* < .01. (H) 3‐HAA but not kynurenine slowed the xenograft growth of PDX HCCs. *n* = 6. (I) The volcano map of the top consistently upregulated genes in SMMC7721, and HepG2. Deep sequencing was used to profile gene expression in HCC SMMC7721, and HepG2 cells. HCC cells were treated with 100 μM 3‐HAA for 8 h. (J) Top consistently upregulated genes were individually verified using quantitative PCR and immunoblotting. (K) DUSP6 expression correlated with overall survival of HCC patients, as analysed by the K‐M plotter. The total patient number was 415. The HCC patients were divided into two groups by the median value of KMO expression. (L) Effects of *DUSP6* knockdown on the growth of HCC cells. Cells were treated with 100 μM 3‐HAA for the indicated time. *DUSP6* was stably knocked down in SMMC7721 cells using lentivirus‐generated shRNA. **: *p* < .01. (M) Effects of DUSP6 knockdown on HCC cell apoptosis. SMMC7721 cells were treated with 100 μM of 3‐HAA for 12 h, stained for Annexin V, and analysed by flow cytometry. *: *p* < .05

Through gene expression profiling, real‐time PCR and immunoblotting, the top two upregulated genes *DUSP6* and *IGFBP1* were selected for further study (Figure 3I,J). However, the clinical data showed that the overall survival of HCC patients was only associated with the expression level of DUSP6, but not IGFBP1 (Figure 3K; Figure S2D). Patients expressing a high level of DUSP6 showed a more prolonged overall survival than patients expressing a low level of DUSP6 (Figure 3K), and the corrective analysis with the clinical characteristics also supported this finding (Figure S2E). Also, we demonstrated that DUSP6 mediates 3‐HAA‐induced tumour cell apoptosis via ERK signalling (Figure 3L,M; Figure S2F,G), which was consistent with our previous finding.^4^

According to the fact that 3‐HAA activates transcription factor YY1,^4^, ^5^ closer analysis of the *DUSP6* promoter region using online‐based prediction tools^6^, ^7^ revealed a novel potential YY1 binding DNA fragment at positions −1145 to −1134, which was distinct from the reported consensuses binding sequence.^8^ This finding was further confirmed by a luciferase assay and ChIP‐QPCR (Figure S2H,I). The TUNEL assay demonstrated that 3‐HAA‐induced apoptosis was reduced in SMMC7721 cells depleted of YY1, overexpression of DUSP6 restored the apoptosis suppressed by YY1 depletion (Figure S2J).

**KMO enhances the inhibition effect of IDO1 inhibitor on HCC growth**. The various HCC mouse models were implemented to further evaluate the potential application of KMO target in clinics. As shown in Figure 4A, DUSP6 knockdown reversed KMO‐mediated suppression of tumour growth in SMMC7721 xenografts. More impressively, KMO overexpression reduced the tumour numbers and prolonged the survival in a transposon HCC mouse model. DUSP6 depletion promoted tumour formation and shorten mice survival, and KMO overexpression had little effect on tumour formation and mice survival after DUSP6 knockdown (Figure 4B).

**FIGURE 4:**
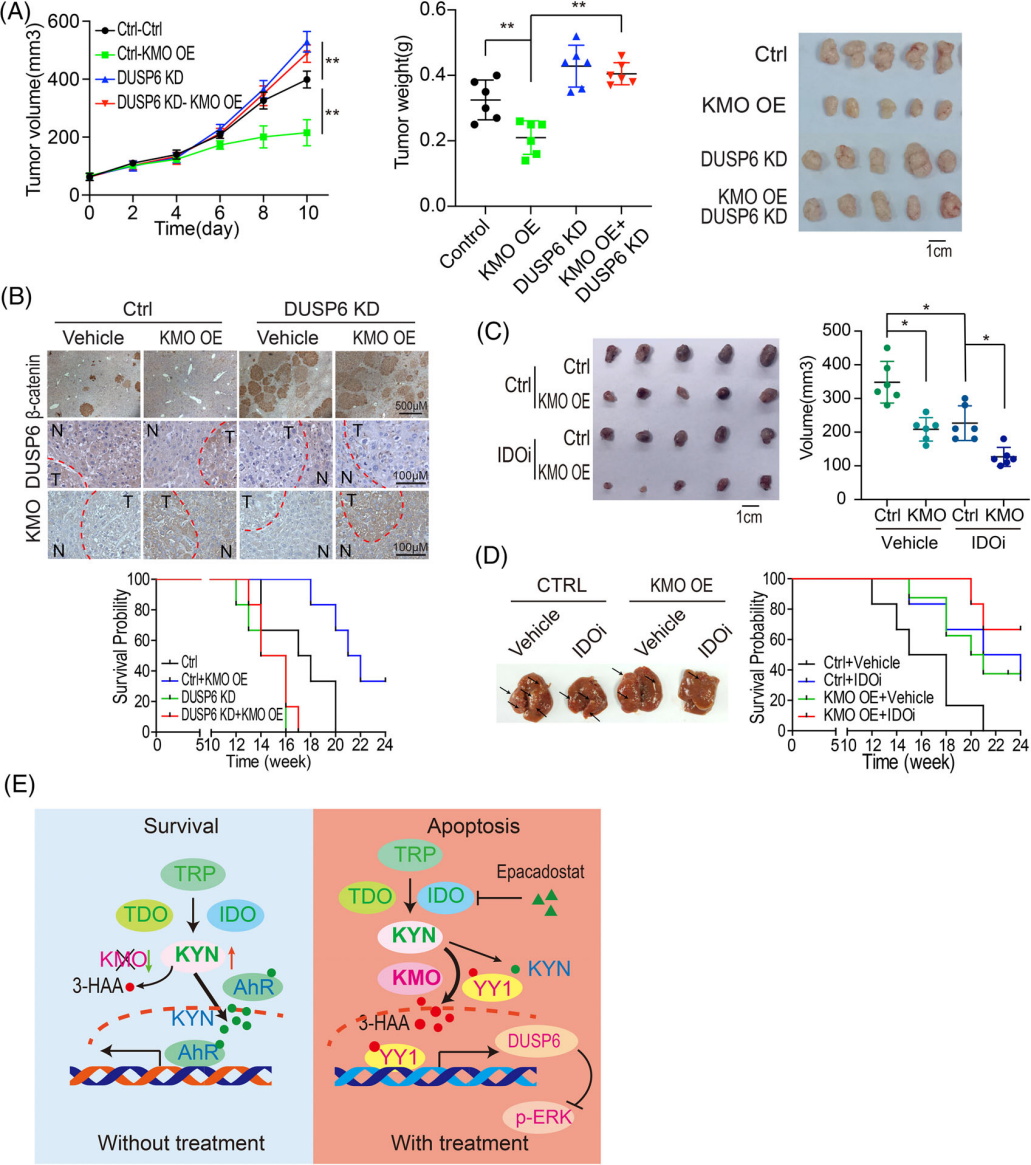
KMO enhances the inhibition effect of IDO1 inhibitor on HCC growth. (A). *DUSP6* knockdown recovered KMO‐suppressed xenograft growth. SMMC7721 cells overexpressing KMO were subcutaneously injected into mice. The middle graph shows xenograft weights in different groups. Photographs on the right show representative tumours in different groups (*n* = 6). **: *p* < .01. (B) *DUSP6* knockdown restored KMO‐reduced tumour numbers and shortened the survival of mice bearing transposon HCCs. The genetic transposon HCC mouse model was established as described in the section of methods and materials (*n* = 6). (C) KMO overexpression enhances the inhibition effect of IDO1 inhibitor Epacadostat to suppress HCC xenograft growth. The mouse liver cancer Hep1‐6 cells (1 × 10^6^) were inoculated into immune‐competent C57BL/6 mice (*n* = 6). *: *p* < .05. (D) KMO overexpression prolongs the survival of mice bearing transposon HCCs with IDO1 inhibitor Epacadostat. The dose of Epacadostat (IDO1 inhibitor) was 100 mg/kg·day. Note: The mouse xenografts were generated by the inoculation of 1.5 × 10^6^ of SMMC7721 cells into the armpit of the rear limb. Tumour volumes are presented as mean ± SD (*: *p* < .05; **: *p* < .01.). Photographs show representative xenografts in different groups. (E) The working model

Most importantly, KMO enhances the effect of IDO1 inhibitor Epacadostat to suppress HCC xenograft growth in an immune‐competent mouse model (Figure 4C). In the meantime, the combination of KMO overexpression with IDO1 inhibitor Epacadostat also inhibited the HCC tumour growth and prolonged the survival of mice bearing transposon‐induced HCCs (Figure 4D).

In brief, this study reveals that both KMO and its substrate 3‐HAA decreases in HCC cells and HCC tissues. The KMO overexpression as well as 3‐HAA treatment reverses the tumour‐promoting effect of kynurenine and significantly improves the efficacy of IDO1/2 inhibitors on HCC xenografts (Figure 4E). These findings show that downregulation of KMO appears to be essential for HCC growth, suggesting the kynurenine metabolic enzyme KMO is a promising therapeutic target for HCC.

## CONSENT FOR PUBLICATION

Not applicable.

## CONFLICT OF INTEREST

The authors declare that they have no competing interests.

## Supporting information

Supporting information
