# Supplementary material for: Kynurenine catabolic enzyme KMO regulates HCC growth
Source: Clin Transl Med. 2022 Feb 20;12(2):e697. doi: 10.1002/ctm2.697 (PMC8858614; doi:10.1002/ctm2.697)
Supplement: Supplementary file 1 — Supporting information [file CTM2-12-e697-s001.pdf]

Figure S1

A

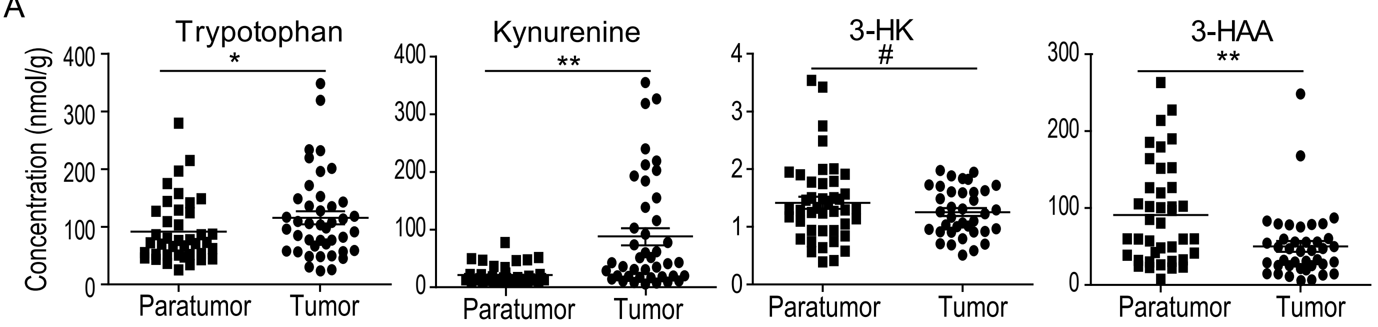

Figure S1 legend

A. The quantitative analysis of general tryptophan catabolites in esophagal carcinoma by mass-spec. The esophageal carcinoma patients were 42 cases. The *: $p<0.05$; the **: $p<0.01$; the #: $p>0.05$.

Figure S2

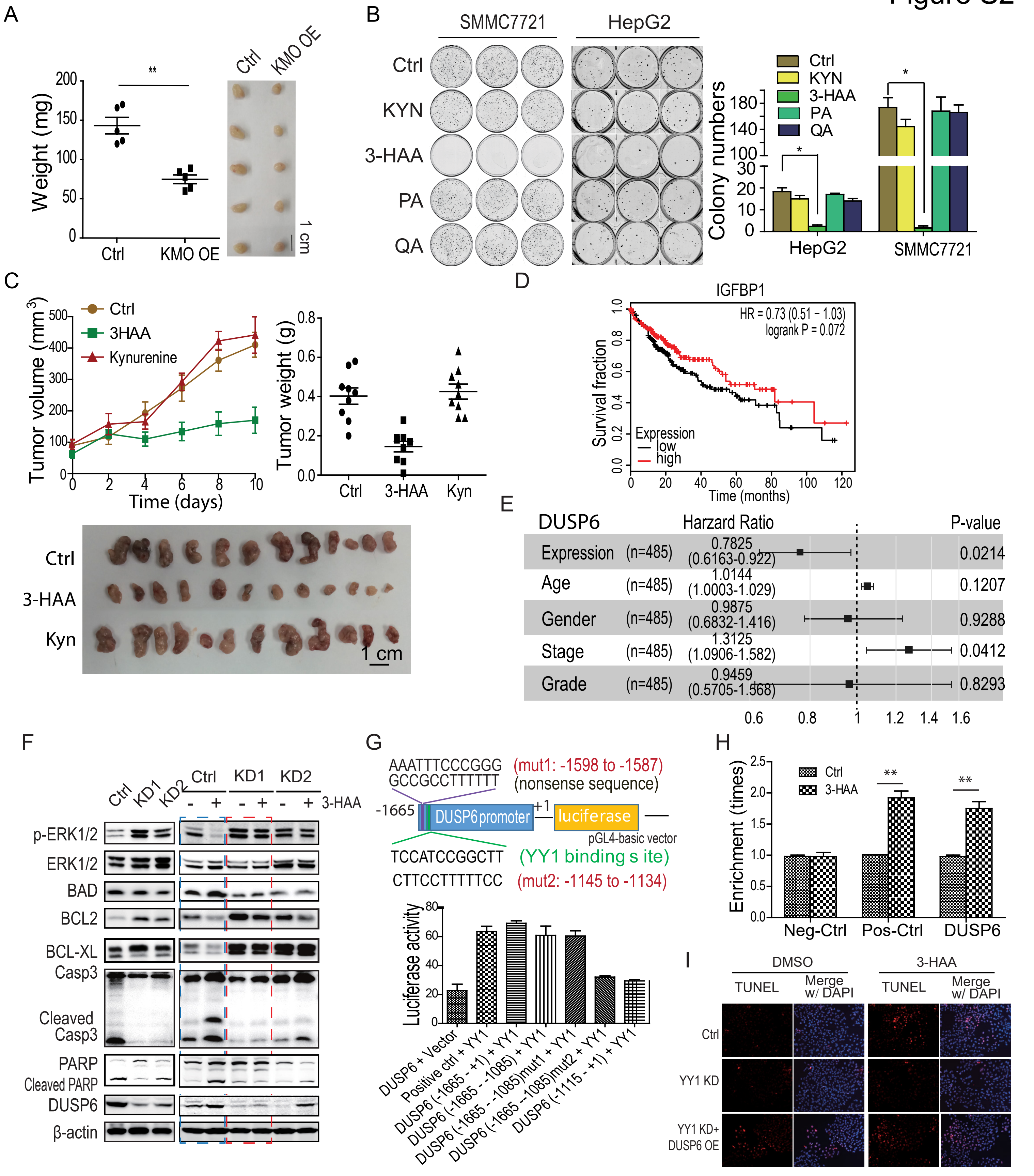
A
Weight (mg)
Ctrl
KMO OE
1 cm
B
SMMC7721
HepG2
Ctrl
KYN
3-HAA
PA
QA
Colony numbers
HepG2
SMMC7721
C
Tumor volume (mm3)
Ctrl
3HAA
Kynurenine
Time (days)
Tumor weight (g)
3-HAA
Kyn
1 cm
D
IGFBP1
HR = 0.73 (0.51 − 1.03)
logrank P = 0.072
Survival fraction
Expression
low
high
Time (months)
E
DUSP6
Harzard Ratio
P-value
Expression (n=485) 0.7825 (0.6163-0.922) 0.0214
Age (n=485) 1.0144 (1.0003-1.029) 0.1207
Gender (n=485) 0.9875 (0.6832-1.416) 0.9288
Stage (n=485) 1.3125 (1.0906-1.582) 0.0412
Grade (n=485) 0.9459 (0.5705-1.568) 0.8293
F
KD1
KD2
3-HAA
p-ERK1/2
ERK1/2
BAD
BCL2
BCL-XL
Casp3
Cleaved Casp3
PARP
Cleaved PARP
DUSP6
β-actin
G
AAATTTCCCGGG
GCCGCCTTTTTT
(mut1: -1598 to -1587)
(nonsense sequence)
-1665
DUSP6promoter
+1
luciferase
pGL4-basic vector
TCCATCCGGCTT
(YY1 binding s ite)
CTTCCTTTTTCC
(mut2: -1145 to -1134)
Luciferase activity
DUSP6 + Vector
Positive ctrl + YY1
DUSP6 (-1665 - +1) + YY1
DUSP6 (-1665 - -1085) + YY1
DUSP6 (-1665 - -1085)mut1 + YY1
DUSP6 (-1665 - -1085)mut2 + YY1
DUSP6 (-1115 - +1) + YY1
H
Enrichment (times)
Neg-Ctrl
Pos-Ctrl
I
DMSO
TUNEL
Merge w/ DAPI
YY1 KD
YY1 KD+ DUSP6 OE

Figure S2 legend

A. The endpoint tumor weight and representative tumors in two groups of cell-derived xenografts.

B. The effect of four tryptophan metabolites on colony formation of HCC cells. The concentration of kynurenine, 3-HAA, picolinate and quinolinate was 100 μM. The cells were treated for 14 days. **: P<0.01.

C. The effects of 3-HAA/kynurenine on the growth of SMMC7721 xenografts. The concentration of both 3-HAA and kynurenine was 100 mg/Kg.Day. *: P<0.05

D. The overall survival analysis of IGFBP1 expression with HCC patient.

E. Corrective analysis on overall survival of HCC patients with clinical characteristics

F. Effects of DUSP6 knockdown on 3-HAA-activated apoptotic signaling. SMMC7721 cells depleted of DUSP6 were treated with 100 μM 3-HAA for 24 h.

G. Transcription activity of YY1 on the DUSP6 promoter, as determined in a luciferase reporter assay. The schematic depicts the plasmid encoding luciferase under the control of the DUSP6 promoter, which was truncated or mutated as indicated.

H. The ChIP-QPCR analysis. The positive control (PC) is TP53. **: P < 0.01.

I. The apoptosis analysis by TUNEL assay in SMMC7721 cells depleted of YY1 and/or overexpressing DUSP6.

Methods and Materials

Cells

Human HCC cell line HepG2 (American Type Culture Collection; Manassas, VA, USA; RRID: CVCL_0027), Hep3B (RRID: CVCL_0326), PLC8024 (RRID: CVCL_0485) (Cell Bank of the Chinese Academy of Science, Shanghai, China), MHCC97L (RRID: CVCL_4973), MHCC97H (RRID: CVCL_4972), Huh7 (RRID: CVCL_0336), LO2 (RRID: CVCL_6926), WRL68(RRID: CVCL_0581) and SMMC7721 (RRID: CVCL_0336) (Genechem Co., Ltd., Shanghai, China) were grown at 37 °C in DMEM (Invitrogen, Grand Island, NY, USA) containing 5% CO_2 atmosphere and supplemented with 10% heat-inactivated fetal bovine serum (PAA, Australia) in the presence of 100 U/mL penicillin and 0.1 mg/mL streptomycin.

Plasmids

The pGIPZ-shHAAO-1, pGIPZ-shHAAO-2, pGIPZ-shHAAO-3, pGIPZ-shDUSP6-1, pGIPZ-shDUSP6-2, pGIPZ-shYY1-1 and pGIPZ-shYY1-2 were purchased from SHSMU DNA Library. The pTSB-KMO was purchased from Genewiz.

Colony formation

HCC cells were seeded into 6-well dishes at a cell density of 1000 cells/well and treated with drugs for 10-14 days until clones were visible. PBS-washed cells were fixed with 4% paraformaldehyde and stained with 1% crystal violet. The stained clones were counted.

Cell proliferation assay

Cell proliferation was measured using the Cell Counting Kit-8 reagent (CCK-8, Dojindo, cat: CK04). HCC cells were seeded into a 96-well plate at 2000 cells/well, were treated with 3-HAA (Sigma, cat: 148776) and ZVAD (TargetMol, cat: T6013) at appropriate doses as indicated in the figure legend. CCK-8 assays were performed in

triplicates as instructed by the manufacturer for 2 hours at indicated time points. Absorbance was measured at 450 nm using a microplate reader, and cell viability was normalized to control, and the mean of at least three independent experiments was calculated.

Flow cytometry

HCC cells were harvested by trypsinization and washed with phosphate-buffered saline (PBS). The cells were then stained with anti-human Annexin V-APC and PI-PE (Multisciences Inc, cat: AP107-100) for 30 min. At least 1 x 10^6 cells were analyzed by a FACS Aria II (BD Falcon, Franklin Lakes, NJ, USA). Cells were gated based on their forward and side scatter properties.

ChIP analysis

Chromatin was isolated from HCC cells treated with or without 3-HAA and fragmented to a size range from 150 to 400 bp. The solubilized chromatin fragments were immunoprecipitated with antibodies against YY1 (Active Motif, cat: 61779).
For ChIP-PCR and ChIP-QPCR, primers (ChIP-NC-F: CACCTGCTTAGCACAGTTTCCA, ChIP-NC-R: GTCCAGCAAAACCTGATGGATT, ChIP-DUSP6-F： CCTCCATCCGGCTTCCAAT, ChIP-DUSP6-R： GGTAACTTTGGGGAGGTGCG, ChIP-PC-F： GACAGGTCTGAAGCCTGGAG, ChIP-PC-R: CGGGACGTGAAAGGTTAGAA) were used to detect the accumulation of YY1 in the DUSP6 promoter.

Western blotting assays

Appropriate cells were lyzed in RIPA lysis buffer containing a cocktail of protease inhibitors (Roche) and PMSF. Total protein concentration was determined using the bicinchoninic acid (BCA) assay kit (Ding Guo Biotechnology, cat: BCA02). For nuclear

and cytoplasmic protein analysis, the Nuclear and Cytoplasmic Protein Extraction Kit (Beyotime, cat: P0028) was used according to the instructions. Antibodies against the following proteins were used for immunoblotting: IDO1 (CST, cat: 86630), TDO2 (Origene, cat: TA504879), KYNU (Proteintech, cat: 11796-1-AP), KMO (Proteintech, cat: 10698-1-AP), HAAO (Proteintech, cat: 12791-1-AP), Cleaved Caspase3 (CST, cat:9315), PARP (Proteintech, cat: 13371-1-AP), DUSP6 (Proteintech, cat: 10433-1-AP), IGFBP1 (Proteintech, cat: 13981-1-AP), p-ERK (CST, cat: 4370), ERK (CST, cat: 4695), BAD (CST, cat: 9239), BCL2 (CST, cat: 4223), BCL-XL (CST, cat: 2764), and β-actin (Santa Cruz, cat: 47778). The immunoblots were scanned using an Odyssey infrared imaging system (LI-COR). Immunolabeling was detected using the ECL reagent (Sigma). Protein expression was normalized against β-actin.

Real-time quantitative PCR

Total cellular RNA was prepared using the TRIzol reagent (Invitrogen, cat: 15596018) as instructed by the manufacturer and was reverse transcribed using a reverse transcription reagent kit (TAKARA, cat: RR036A). After cDNA synthesis, real-time quantitative polymerase chain reaction (PCR) was performed in triplicate in a 96-well plate with an ABI7500 real-time PCR system (Life Technologies, Grand Island, NY, USA) using SYBR Green mixture (AG, cat: AG11702). The primer sequences were as follows: *IGFBP1*-F 5'- GGCTCTCCATGTCACCAACA -3', *IGFBP1*-R 5'- CCATTCCAAGGGTAGACGCA -3', *DUSP6*-F 5'- CCTGCATTGCGAGACCAATC -3', *DUSP6*-R 5'- GGGGGTGACGTTCAAGATGT -3', *NR0B2*-F 5'- GCCTGAAAGGGACCATCCTC -3', *NR0B2*-R 5'- CCAGGGTTCCAGGACTTCAC -3', *EGLN3*-F 5'- CTTTGTGGCCTTCTTTGA AGT -3', *EGLN3*-R 5'- CCACACAGTTGCTCCACAT -3', *PFKFB4*-F 5'- GGATCCCGGACCTCGATTCT -3', *PFKFB4*-R 5'- CCCAGGAAGTTGTCCAGGTAG -3', *IER3*-F 5'-

AAGCCCATCCACCGCTAAAA -3’, *IER3*-R 5’- AGAAGCCTTTTGGCTGGGTT -3’, *β-actin*-F 5’-GCGGGAAATCGTGCGTGACATT-3’ and *β-actin*-R 5’-GATGGAGTTGAAGGTAGTTTCG-3’.

Immunohistochemistry

Tissue samples were fixed in 4% paraformaldehyde (PFA) and embedded in paraffin. Primary antibodies used were 3-HAA (Abcam, ab15580), KYNU (Proteintech, cat: 11796-1-AP), KMO (Proteintech, cat: 10698-1-AP), HAAO (Proteintech, cat: 12791-1-AP), IDO1 (CST, cat: 86630), and DUSP6 (Proteintech, cat: 10433-1-AP). Detection was performed with the Elivision super Kit (MXB, cat: KIT-9921) and DAB Substrate (Boster, cat: AR1022), followed by hematoxylin counterstaining (BBI, cat: E607317).

Dual-luciferase reporter

For luciferase assay, the promoter region of human DUSP6 was moved from pGL2-DUSP6 (a gift from Dr. Norton, University of Florida) by digestion, gel purified, and inserted into a KpnI/XhoI digested pGL4 vector. We constructed some mutants using primers (DUSP6-promoter-F: GGGGTACCCGAACACGCTCCTCCAGG, DUSP6-promoter-R: CCCTCGAGCGGCCGCCAGTGTGAT, DUSP6-F1-m-1598-1587: TGGCAACCTAGGCCTCGCAAAATTTCCCGGGTAGCCTACATTTCCCAAACCAG, DUSP6-R1-m-1598-1587: GGTTTGGGAAATGTAGGCTACCCGGGAAATTTTGCGAGGCCTAGGTTGCCA, DUSP6-F1-m-1145-1134: GGCCCCCACCCGGCCACGCCCTTCCTTTTTCCCCAATCCGTCCGCCCCGCGG and DUSP6-R1-m-1145-1134: GCCGCGGGGCGGACGGATTGGGGAAAAAGGAAGGGCGTGGCCGGGTGGGGGCC).

293T cells were co-transfected with 100ng different pGL4-DUSP6-promoter vectors, 800ng pSG5-YY1, or pSG5, and 1 ng pRL-TK-Renilla luciferase plasmids. After 48

hours, the cells were lysed, and luciferase activity was measured using the Dual-Luciferase Reporter Assay System (Promega) according to the manufacturer's instructions.

RNA-Seq

RNA was extracted from HCC cells with the TRIzol reagent (Invitrogen, cat: 15596018) according to the instructions. RNA-seq libraries were prepared with the Ovation RNASeq Systems 1-16 (Nugen), and indexed libraries were multiplexed in a single flow cell and underwent 75 base pair single-end sequencing on an Illumina NextSeq500 using the High Output kit v2 (75 cycles) at BGI Group.

Lentivirus Production

293T cells were cultured in DMEM supplemented with 10% fetal bovine serum (Gibco) and were maintained at 37℃ in a humidified atmosphere with 5% CO_2. For virus production, 8 μg of the appropriate plasmid and 3.2 mg of helper plasmids (2μg pMD2.G and 6μg psPAX2) were transfected into 293T cells cultured at 80% confluence in a 10 cm dish using Lipoplus (SAGE) according to the manufacturer's instructions. Viral supernatants were collected 48 hours after transfection and filtered through a 0.45 mm filter.

Metabolism flux analysis by LC-MS/MS

For the flux experiment of tryptophan catabolism, tryptophan in the medium was replaced by fully ^{13}C-labeled tryptophan (tryptophan-$^{13}C_{11}$). Sample preparation and LC-MS/MS analysis were the same as for quantitation of tryptophan catabolic products, except that the parameters of MRM transitions were different. MRM transitions for catabolic products of tryptophan-$^{13}C_{11}$ (M11: 320 > 273) were set as 5-hydroxyindoleacetic acid (M10: 323 > 155), kynurenine (M10: 427 > 122), kynurenic acid (M10: 304 > 105), 3-hydroxykynurenine (M10: 547 > 05), xanthurenic acid (M10: 424 > 05), 3-hydroxyanthranilic acid (M7: 369 > 247), cinnabarinic acid (M14: 419 > 105).

Simultaneously, the MRM transitions (M0) of unlabeled tryptophan and its catabolic products were acquired using the same settings as in quantitation experiments.

TUNEL assay

The cover glass was placed in the 24-well plate, and the HCC cells were inoculated on the cover glass for 48 hours. The cells were washed with PBS for three times. Add 0.5mL of 4% paraformaldehyde and fix cells at room temperature for 10 minutes. Cells were treated with 0.4%Triton X-100 for 5 minutes and washed with PBS. Cells on the cover glass were treated with TUNEL staining solution and incubated in a wet box at 37℃ for 1 hour. DAPI staining solution was used to stain the nuclear for 5 minutes in dark. Cells were observed and photographed under a fluorescence microscope.

HCC-PCX mouse model

Six-week-old male BALB/c nude mice or male immune-competent C57BL/6 mice were purchased from Lingchang, Shanghai, China. Xenograft mouse model of HCC was generated by injecting SMMC-7721 HCC cells (1.5×10^6) or mouse live cancer Hep1-6 cells (1 × 10^6) subcutaneously into the armpit of the rear limb. These HCC cells were overexpressing KMO or depleted of DUSP6. The IDO1 inhibitor Epacadostat was orally administered at the dose of 100 mg/kg.day. After two weeks, subcutaneously transplanted tumors were removed, and the volume was measured, and the tumors were photographed.

HCC-PDX mouse models

This study received ethics board approval at the Shanghai Jiao Tong University School of Medicine. The HCC-PDX models (LIV#031, #046, and #057) were initially isolated from patients and were stored in liquid nitrogen. Mice were maintained under specific-pathogen-free (SPF) conditions. Once the recovered tumors grew to 250 mm^3 in mice, tumor tissues were cut into 2×2 mm pieces and

implanted subcutaneously into SCID mice [13]. The 3-HAA were intraperitoneally administered every day when the tumor volume reached approximately 200 mm^3. Tumor size and mice body weight were monitored for up to 4 weeks, and tumor volume (TV) was calculated.

Transposon HCC mouse Model

This induced HCC mouse model was adopted from the literature [14-16]. Briefly, HCC inducing oncogenes β-Catenin and MET in pT2 vector along with Sleeping Beauty transposon (SB100) was introduced with GFP, pT2-shDUSP6, or pT2-shYY1 (also in pT2 vector) into C57BL/6 mice. Thirty micrograms of the oncogene plasmids and three micrograms SB100 plasmids were diluted in 2 ml of a filtered 0.9% NaCl solution and followed by an injection into the lateral tail veins of 6-week-old mice. Livers of some mice were harvested to determine tumor burden at a specific time after hydrodynamic transfection (HDT). The six mice in each group were used for survival analysis.

Statistical analysis

Data were presented as means ± SD. All data were representative of at least three independent experiments. The unpaired two-tailed Student's *t*-test and the Two-way ANOVA were used as indicated. All presented differences were $P < 0.05$ unless otherwise stated.
